# Data for TROTS – The Radiotherapy Optimisation Test Set

**DOI:** 10.1016/j.dib.2017.03.037

**Published:** 2017-04-01

**Authors:** Sebastiaan Breedveld, Ben Heijmen

**Affiliations:** Erasmus University Medical Center – Cancer Institute, Department of Radiation Oncology, Rotterdam, The Netherlands

**Keywords:** 90C06, 90C26, 90C29, 90C30, Radiotherapy, Nonlinear optimisation, Multiple objective programming, OR in health services, Large-Scale Optimisation

## Abstract

The Radiotherapy Optimisation Test Set (TROTS) is an extensive set of problems originating from radiotherapy (radiation therapy) treatment planning. This dataset is created for 2 purposes: (1) to supply a large-scale dense dataset to measure performance and quality of mathematical solvers, and (2) to supply a dataset to investigate the multi-criteria optimisation and decision-making nature of the radiotherapy problem. The dataset contains 120 problems (patients), divided over 6 different treatment protocols/tumour types. Each problem contains numerical data, a configuration for the optimisation problem, and data required to visualise and interpret the results. The data is stored as HDF5 compatible Matlab files, and includes scripts to work with the dataset.

## Specifications Table

**Table:** 

Subject area	Medicine, Operational Research, Numerical and Multi-Criteria Optimisation
More specific subject area	Radiotherapy (Radiation Therapy)
Type of data	Numerical data (pencil-beam dose matrices), problem description, patient data
	(computer tomography (CT) scans, delineations of anatomical structures), scripts
How data was acquired	Simulated dose computation on anonymised CT scan
Data format	Raw and Analysed, in HDF5 compatible Matlab files
Experimental factors	
Experimental features	
Data source location	Erasmus University Medical Center Rotterdam, The Netherlands
Data accessibility	Data is publicly available on our website:
	http://www.erasmusmc.nl/radiotherapytrots

## Value of the data

• The data can be used to evaluate performance and quality of (general) mathematical solvers
• The data can be used to compare different solvers in general or those used in radiotherapy treatment planning
• The data can be used to investigate different multi-criteria optimisation and decision-making approaches in radiotherapy
• The data can be used by groups who want to extend their research interests to radiotherapy, but do not have access to this type of medical data

## 1 Data

When a patient is diagnosed with cancer and selected for treatment with radiotherapy, a *treatment plan* has to be generated. This is based on a 3D Computer Tomography (CT) scan of the patient, containing delineations of the organs and the tumour. The treatment plan describes the personalised settings of the applied treatment unit, and contains a predicted patient dose distribution for these settings, projected on the CT-scan. The aim is to deliver sufficient dose to the tumour for curation, while keeping the dose to healthy organs as low as possible to minimise the probability of developing radiation-induced treatment related complications.

Computing a treatment plan is a large-scale nonconvex nonlinear combinatorial multi-criterial optimisation problem, to be solved within a limited time-frame, and to acceptable optimality (otherwise the patient might not be treated as well as technically possible). As each patient is anatomically unique, the treatment planning process (optimisation and decision-making) has to be performed for each individual patient.

The data provided allows to investigate two applications: (1) For a chosen problem definition, the performance and accuracy for mathematical solvers can be evaluated, irregardless of the clinical interpretation of the result (see [1]). (2) For multi-criteria optimisation and decision-making (MCDM), different clinical trade-offs can be investigated, irregardless of the performance of the mathematical solver (see [2]).

More information on the technical background of radiotherapy treatment planning can be found in [3], [4], and on the use of the data can be found in [1], [2], [5].

## 2 Experimental design, materials and methods

This dataset contains data required for radiotherapy treatment plan optimisation for 120 patients which were treated previously at the Erasmus University Medical Center Rotterdam, The Netherlands. The patients belong to different groups of tumour locations, tumour types and types of treatment, and were included randomly in the original studies, see Table 1. For the Head-and-Neck patients, we included an alternative set for the same 15 patients with a more accurate dose model. This results in denser matrices, and thus a heavier problem from a numerical perspective. Because the problem complexity between the two sets is comparable, this allows evaluating the impact on the numerical performance of mathematical solvers.

**Fig. 1:**
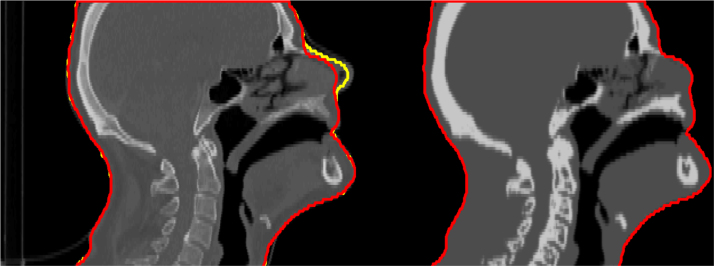
Anonymisation of CT. The left panel shows original CT with original patient body contour in yellow. Both the original CT and body contour could be abused to reconstruct the facial profile. The red contour smooths distinctive properties of the nose, forehead and mouth. To further prevent reverting the anonymisation process, the CT is flattened (right panel).

We refer the reader to the references given in the table for more background on the supplied data. For this dataset, no personal information of the patients is stored or required, with the exception of the CT data. To avoid potential facial recognition, the facial profile of head-and-neck patients is smoothed, and the grey levels of the CT are flattened, see Fig. 1.

Due to the nonconvexity of the radiotherapy treatment planning problem (see [2]), some *a priori* choices were made in generating this dataset using the methods described by the references in Table 1. This includes (among others) selection of treatment beam geometry, proton spot positions and (proton) energy layers. Consequently, these choices may not be optimal for structurally different multi-criteria optimisation choices, especially for the *Protons* plans.

**Fig. 2:**
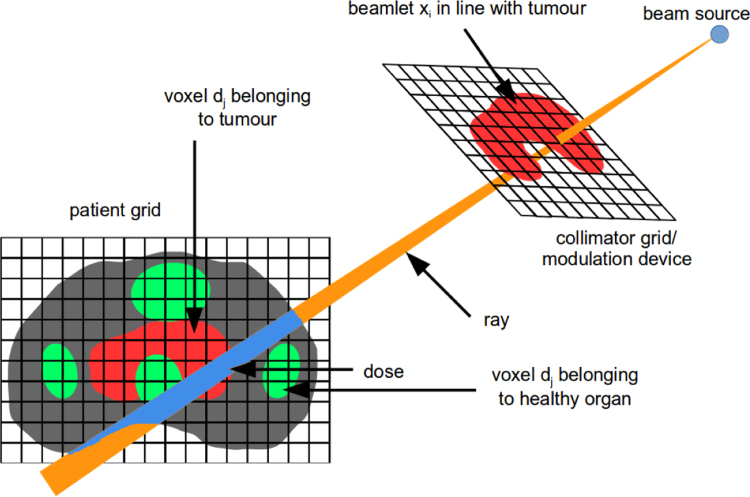
Radiotherapy problem decomposition. Ionising radiation originates from the beam source point and falls onto a collimator. This device allows shaping the beam in different forms and intensities, and is discretised in *beamlets*. The longer a beamlet is “open”, the higher the intensity through that beamlet, and the higher the resulting dose in the patient. As soon as the *pencil-beam* enters the patient, the ionising radiation interacts with the tissue, leading to dose (cell damage), measured in *Gray (Gy)*. The patient is discretised in *voxels*. (Figure from [18]).

For each patient, the data is prepared according to the following workflow:

1. acquire CT scan
2. delineate tumour(s), organs and other volumes of interest on the CT (see Fig. 2)
3. anonymise patient׳s facial profile (see Fig. 1)
4. define treatment protocol (see Table 1)
5. convert treatment protocol to (personalised) mathematical multi-criterial optimisation problem
6. compute pencil-beam dose matrices required for treatment plan optimisation [15], [16]
7. solve multi-criterial problem using methods described in [12], [14], [17]
8. based on this result, the optimisation problem is rewritten to an equivalent weighted-sum optimisation problem using [17]
9. this optimisation problem, matrices (mainly the pencil-beam dose matrices) and patient data required for visualisation of the data defines the data in this set

Step 8 is introduced to simplify the evaluation of mathematical solvers. This weighted-sum problem contains the correct weights to give the identical result of a full multi-criteria optimisation, leaving a simple single run with a sane result from a radiotherapy perspective.

### 2.1 Data format

This section describes the contents of the files. The data is stored in *Matlab* files, MAT version 7.3. The files are fully HDF5 compliant, and can therefore be read using general HDF5 tools.

Each file contains 3 structures: **problem** defining the mathematical optimisation problem, **data** containing the numerical data matrices, and **patient** containing the CT scan and other information required for visualisation of the data. There is also a **solutionX** vector containing the solution to the standard problem, as defined by the papers and optimisation methods referenced in Table 1. The *Prostate VMAT* and *Head-and-Neck* groups also have an alterative solution **solutionX**_**alt**, which was obtained by using a different solution strategy (see [9], [11]). In this paper we only describe the most relevant items of the data format, the detailed description can be found in a document as part of the dataset [19].

To better understand the notation used in this section, a short technical background of the radiotherapy problem is visualised in Fig. 2. The optimisation problem optimises the decision variables *x*, representing the intensities of the *pencil-beams*. The relation to the dose in the patient is linear: *d*=*Ax*, where *d* is the discretised dose in the patient, and *A* is called the pencil-beam matrix. To optimise on different organs (volumes of interest on the CT) separately, each organ has its own pencil-beam dose matrix *A* in the **data** structure, on which one or more constraints/objectives can be imposed as defined by the **problem** structure.

#### 2.1.1 The **problem** structure

The **problem** structure is a list, where each entry defines an objective or constraint. Each entry has the following fields:

• **dataID** Reference index to the **data** structure, containing the respective numerical data.
• **Name** A name that refers to the clinical structure this constraint/objective is based on. Is ignored by the solver.
• **Minimise** If *True* for an *objective*, this objective will be *minimised*. If *True* for a *constraint* this is a *maximum* constraint. If set *False*, vice versa.
• **Type** Identifier for the used *cost-function* (see document in dataset [19]).
• **Parameters** Sets parameters to configure the *cost-function*, given in **Type**.
• **Objective** For a *constraint*, this is the value the cost-function is constrained to. For an *objective* in a multi-criteria setting, this is the aspired value.
• **Sufficient** For an *objective* in a multi-criteria problem, the objective value does not need to become lower (higher) than the given sufficient value. Is ignored when set empty.
• **Weight** Scalar to apply to the *objective*, useful to scalarise and weigh multiple objectives.
• **Priority** Natural number that indicates the priority of this *objective*. Used in multi-criteria optimisation.
• **Active** Can be *True* or *False* to enable or disable this objective/constraint.
• **IsConstraint** If *True*, this entry is a *constraint*, and an *objective* otherwise.
• **Chain** Extra information for *chain* function type (see document in dataset [19]).

For a mathematical solver, only the entries *dataID*, *Minimise*, *Type* (together with *Parameters* and *Chain*), *Objective* (when *IsConstraint* is true), *Weight*, *IsConstraint* and *Active* are relevant.

For a multi-criteria optimisation, the entries *Priority*, *Objective* (for objectives), and *Sufficient* define the relative importances and aspirations for the objectives. These are directly derived from the automated treatment planning configuration, see [14], [17] and the references given in Table 1.

#### 2.1.2 The **data** structure

The data structure contains 2 substructures: *matrix*, containing the numerical data, and *misc*, containing auxiliary data to configure the problem (see [1], [19] for details). In this paper, we only describe the *matrix* substructure, which has the following fields:

• **Name** A name that refers to the clinical volume (e.g. organ name or the name given to the artificial structure), or other background of this data.
• **A** The data *matrix*. Each matrix in the **data** structure has an equal number of columns, equal to the number of decision variables. In radiotherapy, this matrix is generally the *pencil-beam* dose matrix. The number of rows typically indicate the number of voxels (sampled elements in the CT where the dose is evaluated), and the number of columns equals the number of pencil-beam weights.
• **b** Offset *vector*, is 0 unless you are doing something exciting such as generating a treatment plan on top of an already delivered dose.
• **c** A *scalar* for quadratic cost-functions, empty otherwise.
• **Type** Indicating the matrix type. When *Type=0*, this is a “normal” matrix operating in the *fluence-to-dose* domain, where the argument *d* for the cost-functions is computed as d=Ax+b. *Type=1* indicates a non-dose matrix, but is treated equally as *Type=0* by mathematical solvers. *Type=2* indicates a *quadratic* or *square* matrix.

#### 2.1.3 The **patient** structure

The patient structure is not required for optimisation, but useful in visualisation and interpretation of the results. Information on usage can be found in the scripts (Section 2.2).

### 2.2 Scripts

The dataset is accompanied with Matlab scripts to read, interpret and visualise the data. For more details, see the help section in these functions.

• **TROTSReadOutput** Reads solution vector from textfile as returned by [1].
• **TROTSShowSolution** Shows the requested and attained values for the objectives and constraints side-by-side.
• **TROTSViewDVHs** Shows dose-volume histograms for the solution.
• **TROTSComputeDose** Computes a 3D dose distribution from the numerical solution. This dose distribution is an interpolation of the (known) dose delivered to the sampled points used for plan optimisation.
• **TROTSViewPatient** Interactive viewer to view the CT of the patient, optionally overlayed with the 3D dose distribution in dose-wash or isodose mode. The user can switch between axial, coronal and sagittal cross sections.

## Disclaimer

The provided data originates from research and is for research purposes only. No patients were actually treated using the solutions (dose distributions/treatment plans) resulting from this dataset. Individual solutions were also not verified by physicians (medical doctors). Although most of the provided treatment protocols have a basis in protocols used in our clinic, deviations from the clinical protocol may occur.

## Figures and Tables

**Table 1:** Background of patients.

Identifier	Number	Description	Background
Prostate CK	30	Prostate cancer patients treated with a protocol for inhomogeneous tumour prescription, using 25 beam directions.	[6], [7]
Prostate VMAT	30	Prostate cancer patients for 3 different prescriptions (prostate only, prostate and seminal vesicles with 2 dose levels, prostate and seminal vesicles with same dose), to be treated with Volumetric Modulated Arc Therapy (VMAT).	[8], [9]
Head-and-Neck	15	Patients with cancer in the head-and-neck region, to be treated with VMAT.	[10], [11]
Head-and-Neck Alt	15	Same patients as *Head-and-Neck*, but with denser pencil-beam dose matrices.	
Protons	20	Patients with cancer in the head-and-neck region, treated with 3-beam proton therapy.	[12], [13]
Liver	10	Liver cancer patients, with nonconvex cost-functions, treated with 15 beam directions.	[14]
